# Clinical and demographic characteristics of glaucoma patients in Kingston, Jamaica

**DOI:** 10.1007/s10792-026-04020-y

**Published:** 2026-04-29

**Authors:** Briana L. McIntosh, Osahon J. Asowata, Kaitlyn L. Funk, Kendall R. Pixley, Camara S. Brown, Jessica N. Cooke Bailey

**Affiliations:** 1 Center for Health Disparities, Department of Pharmacology & Toxicology, Brody School of Medicine, East Carolina University, Room 6S10, 600 Moye Blvd, Greenville, NC 27834 USA; 2 University of Cincinnati College of Medicine, 3230 Eden Ave, Cincinnati, OH 45267 USA; 3 Kingston Public Hospital, North Street, Kingston, Jamaica; 4 Department of Population and Quantitative Health Sciences, Cleveland Institute for Computational Biology, Case Western Reserve University, 10900 Cornell Ave, Cleveland, OH USA

**Keywords:** Primary open-angle glaucoma, Optical coherence tomography, African Caribbean population

## Abstract

**Purpose:**

To characterize the clinical, demographic, and imaging features of glaucoma among adults receiving care at Kingston Public Hospital (KPH) Ophthalmology clinic, Jamaica’s largest public eye care facility.

**Methods:**

We conducted a retrospective, cross-sectional chart review of adult glaucoma patients seen at the KPH Ophthalmology Clinic between January 2018 and March 2023. Included patients had documentation of at least two comprehensive ophthalmic examinations and one high-quality optical coherence tomography (OCT) scan (signal strength ≥ 6). Extracted data included demographics, intraocular pressure (IOP), visual acuity, OCT-derived optic nerve head and retinal nerve fiber layer (RNFL) parameters, visual field (VF) metrics, glaucoma severity, comorbidities, and self-reported treatment adherence. Comparisons were performed by age group (< 40 vs. ≥ 40 years) and sex.

**Results:**

A total of 324 patients (619 eyes) were included (mean age 58.3 ± 12.3 years; 66.0% female). At presentation, 51.0% had advanced-stage glaucoma, and only 17.3% reported consistent medication use. Hypertension and diabetes were common comorbidities (80.3% and 38.8%, respectively). Mean Goldmann IOP was 22.0 mmHg, mean RNFL thickness was 75.2 ± 14.9 μm, and mean cup-to-disc ratio was 0.73 ± 0.10. VF indices demonstrated moderate functional loss, with worse mean deviation among men. Longitudinal VF data were limited; only 17% of patients had more than one documented test, limiting cohort-wide assessment of functional progression.

**Conclusion:**

This clinic-based characterization of glaucoma in Jamaica reveals a high burden of advanced disease at presentation, low treatment adherence, and gaps in longitudinal monitoring, supporting the need for prospective studies to develop ancestry—and region-specific OCT reference data and improve equitable glaucoma care.

**Supplementary Information:**

The online version contains supplementary material available at 10.1007/s10792-026-04020-y.

## Introduction

Glaucoma is a multifactorial optic neuropathy and a leading cause of irreversible blindness worldwide[1, 2]. Characterized by progressive structural damage to the optic nerve head (ONH) and thinning of the retinal nerve fiber layer (RNFL), glaucoma often advances asymptomatically until substantial and permanent vision loss has occurred [3, 4]. Despite advances in treatment, early detection and timely intervention remain challenging, particularly among populations at highest risk and those with limited access to eye care services [5]. Globally, the highest glaucoma prevalence rates are seen in Africa and among people of African descent, particularly those of West African and African Caribbean backgrounds [6]. Populations of African descent experience a disproportionate burden of glaucoma, characterized by higher prevalence, earlier onset, and more severe disease compared with Latinx and white populations [3, 6–8]

Beyond differences in prevalence, populations of African descent exhibit distinct biological and anatomical characteristics and face social and structural factors that together complicate glaucoma detection and management [7, 9, 10]. Established risk factors for glaucoma include elevated intraocular pressure (IOP), age (≥ 40 years), genetic predisposition, race/ethnicity, and positive family history [8, 11]. Anatomical and physiological features, including larger ONH, thinner central corneas, and increased scleral strain, may contribute to the elevated risk and severity observed in these groups [12–14]. Socioeconomic factors and structural inequalities further influence access to timely diagnosis and ongoing care, contributing to disparities in glaucoma outcomes [2, 7, 15].

Limitations in current diagnostic tools also play a role in these disparities. Ocular coherence tomography (OCT) is central to contemporary glaucoma diagnosis and monitoring; however, many OCT derived assessments rely on normative databases that remain largely composed of a populations of European or Asian Ancestry[16]. This lack of ancestral representation may reduce diagnostic validity by failing to account for anatomical and risk heterogeneity, particularly among populations of African descent [17]. As a result, disease severity may be underestimated or misclassified, especially in early stages of glaucoma when intervention is most effective [18]. Addressing this gap is critical for improving diagnostic accuracy and supporting equitable treatment strategies [17, 19].

Jamaica represents a critical yet understudied population in this context. As the largest English-speaking Caribbean country, Jamaica is home to nearly three million people, over 90% of whom are of African genetic ancestry, and is genetically distinct from African and other Caribbean nations due to unique historical and migratory influences [20–22]. Consistent with this history, Jamaicans generally have a higher proportion of African ancestry than African Americans [20]. Jamaica also has a large diaspora exceeding two million in the United Kingdom, United States (US), Canada, and other Caribbean countries, extending the relevance of Jamaica-specific clinical data beyond the island itself [23, 24]. Although Jamaica is classified as an upper-middle income country with a relatively well-structured public healthcare system that offers universal access to many services [23], significant challenges persist in the management of chronic diseases, including those related to eye health and vision care [25, 26]. Gaps in Jamaica’s eye care system contribute to delayed glaucoma diagnoses, advanced disease at presentation, and high rates of preventable vision loss [27, 28]. While national prevalence estimates are outdated [29, 30], local ophthalmologists report increasing disease severity, especially among underserved populations [31]. Taken together, Jamaica’s unique genetic ancestry profile, large African-Caribbean diaspora, and the absence of modern imaging-based glaucoma characterization studies underscore the country’s importance as a high-value and underrepresented context in global glaucoma research.

Population-based studies across Africa and the Caribbean further contextualize these observations, consistently demonstrating both a high prevalence of glaucoma and a tendency toward advanced disease at presentation [2, 6, 32, 33]. Surveys in sub-Saharan Africa have reported glaucoma prevalence rates ranging from approximately 4–8%, with nearly half of cases previously undiagnosed [34, 35]. Similarly, in the Caribbean, the landmark Barbados Eye Study found that open-angle glaucoma affected roughly 7% of adults aged 40 years and older, with rates nearly five times higher than observed in white populations [32]. Studies from St. Lucia and among African-Caribbean populations in the United Kingdom have documented elevated prevalence, strong familial aggregation, and high rates of undetected disease [36, 37]. More recent analyses consistently demonstrate that individuals of African ancestry, both on the African continent and in the diaspora, present at younger ages and with more severe optic nerve damage compared with populations of European ancestry[38].

To address these gaps, this foundational study aims to characterize glaucoma at Jamaica’s largest public hospital, identify gaps in care delivery and monitoring, and establish the groundwork for prospective studies that will develop ancestry-specific OCT normative data to improve diagnostic precision in Caribbean populations.

## Subjects and methods

### Ethical approval

This study was approved by the Southeast Regional Health Authority (SERHA) of the Jamaican Ministry of Health and Wellness, Case Western Reserve University’s Institutional Review Board (STUDY-2020-1648), and East Carolina University’s Institutional Review Board (UMCIRB 24–000234). All data were de-identified prior to analysis, and the study complied with the tenets of the Declaration of Helsinki. Given the retrospective nature of the study and use of de-identified data, informed consent was waived.

### Study design

This retrospective chart review was conducted at KPH, a Type A multidisciplinary acute care facility located in Kingston, Jamaica. As the largest tertiary referral center in the country, KPH serves approximately 67% of the national population and hosts the largest ophthalmology clinic in the English-speaking Caribbean [39]. The clinic manages approximately 200 patients per day. Records are maintained on paper.

### Study sample

Eligible patients were adults aged ≥ 18 years with a confirmed clinical diagnosis of glaucoma documented in the KPH Ophthalmology clinic. Inclusion criteria required availability of (i) at least one high-quality OCT ONH RNFL scan with a signal strength (SS) ≥ 6 in one or both eyes, (ii) documentation from a minimum of two comprehensive clinical encounters that recorded IOP and cup-to-disc-ratio (CDR) measurements. Patients were not excluded based on monocular status, refractive error, prior history of intraocular surgery, ocular or systemic comorbid conditions, or current use of medications.

### Sampling method, data collection

**Fig. 1:**
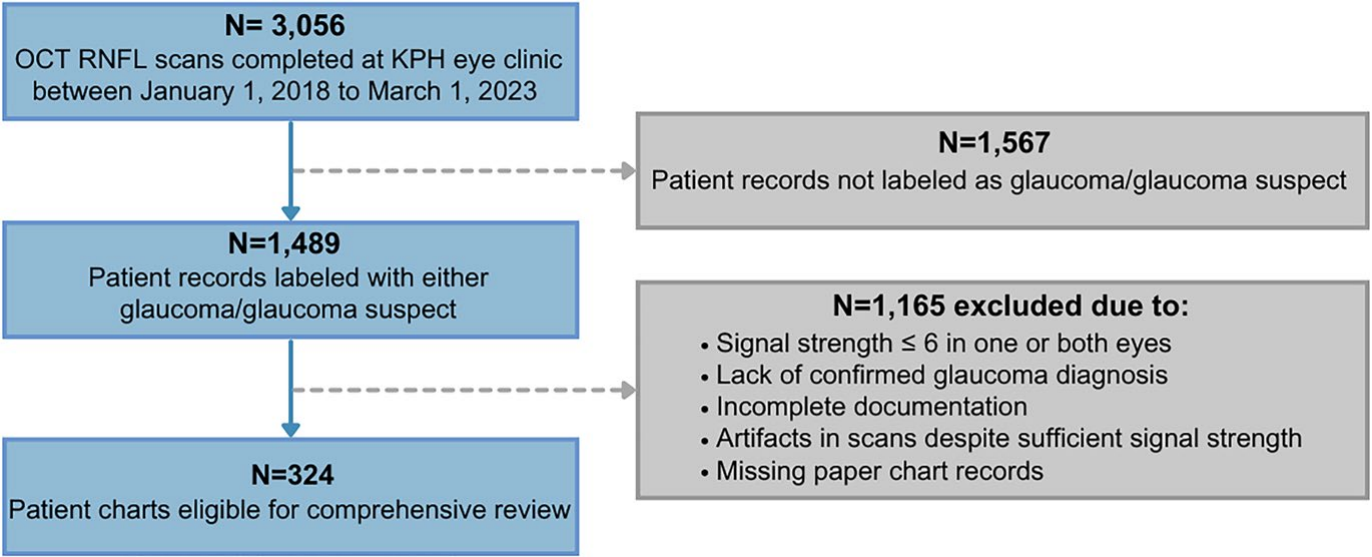
Sampling flowchart for preliminary analyses at the Kingston public hospital (KPH) ophthalmology clinic. Flow diagram illustrating inclusion and exclusion criteria and derivation of the final study sample (n = 324)

A non-probability, clinic-based sampling strategy within a tertiary referral setting was used to identify eligible charts. The OCT ledger in the KPH Ophthalmology clinic was reviewed to identify ONH RNFL 200 × 200 Optic Disc Cube scans performed between January 2018 and March 2023 that were labeled “glaucoma” or “glaucoma suspect.” All OCT ONH-RNFL scans were obtained from a Cirrus HD-OCT Zeiss 5000 [40] machine located in the KPH Ophthalmology clinic. Patient details, including medical record number, name, clinic date, sex, age, scan type, and diagnosis, were recorded in the OCT ledger maintained in the OCT examination room. Scans with large artifacts or SS < 6 were excluded. Corresponding patient charts were retrieved and reviewed for completeness and confirmation of diagnosis. Of 1,489 ledger entries, 621 scans met imaging quality criteria, and 324 corresponding charts contained sufficient clinical documentation for inclusion in the final analytic dataset (Fig. 1).

### Chart review and clinical data extraction

Medical charts corresponding to the 324 eligible glaucoma patients were reviewed from initial referral documentation through the most recent clinic visit. Comprehensive ophthalmologic evaluations at KPH Ophthalmology clinic typically included visual acuity (VA) testing, slit-lamp biomicroscopy, IOP measurements using Goldmann applanation and non-contact tonometry (GAT, NCT, respectively), OCT scans, and, when available, visual field (VF) testing. Eye-specific findings were recorded for the right eye (OD), left eye (OS), and both eyes (OU). In addition to these clinical assessments, chart-based variables including family history of glaucoma, comorbid conditions (e.g., hypertension, diabetes), and other available social or lifestyle factors were recorded. For patients with multiple qualifying OCT scans, the most recent OCT scan was selected for analysis. Trained research staff de-identified all charts, uploaded them to a secure cloud-based system, and entered demographic and clinical variables into a standardized spreadsheet, which was cross-verified for accuracy and imported into IBM SPSS Statistics (Version 29.0.2) for analysis.

### Data collection instruments and definition of variables

#### OCT-derived variables

Key imaging variables were obtained from each OCT scan, including SS, RNFL thickness, RNFL symmetry, CDR, rim area (RA), disc area (DA). SS reflects the quality of the OCT ONH-RNFL scan and is determined by the Signal-to-Noise (SNR) ratio and signal uniformity [41]. RNFL thickness, measured in micrometers (µm), captures the thickness of the retinal nerve fiber layer across a full 360° scan [4]. RNFL measurements were summarized globally and by quadrants: Superior (S), Nasal (N), Inferior (I), and Temporal (T), collectively referred to as SNIT quadrants. RNFL symmetry quantifies inter-eye symmetry, with greater asymmetry suggesting glaucomatous damage or progression [4]. CDR was extracted from ONH measurements; vertical CDR (VCDR) was defined as the ratio of the vertical diameter of the optic cup to that of the optic disc [42]. Additional OCT variables, RA and DA, were used to further characterize ONH structure [43].

#### Clinical Chart Variables

Non-imaging variables abstracted from patient charts included VA, IOP, GAT, NCT, glaucoma diagnosis, glaucoma type, disease severity, family history of glaucoma, trauma history, relevant systemic and ocular comorbidities, and treatment history. History of ocular trauma was recorded as a broad, chart-documented historical variable and was not used to define glaucoma subtype; all patients included in the analytic cohort were clinically diagnosed and managed as POAG. Data were collected across at least two clinical encounters to ensure reliability. Glaucoma status and severity were reviewed and confirmed by KPH’s Glaucoma Lead Ophthalmologist based on clinical chart record and OCT findings. Disease staging was conducted in accordance with the Global Glaucoma Staging System (GGSS) [44], while diagnostic criteria followed American Academy of Ophthalmology Preferred Practice Guidelines[45].

### Statistical Analysis

Demographic and clinical characteristics were summarized in appropriate tables. Continuous variables were described using mean (standard deviation), and categorical variables were summarized as counts (percentages). Independent-sample *t*-tests (or the Wilcoxon signed-rank test, reported with median and interquartile range (IQR, for non-normally distributed variables) and Chi-square (*χ*^2^) tests were used, as appropriate, to compare demographic, clinical, and imaging characteristics between sex and age groups (younger [< 40] and older [≥ 40] patients), depending on whether variables were continuous or categorical. Statistical significance was defined as a P < 0.05. All statistical analyses were conducted using R Studio (version 4.4.1)[46].

## Results

### Patient characteristics

**Fig. 2:**
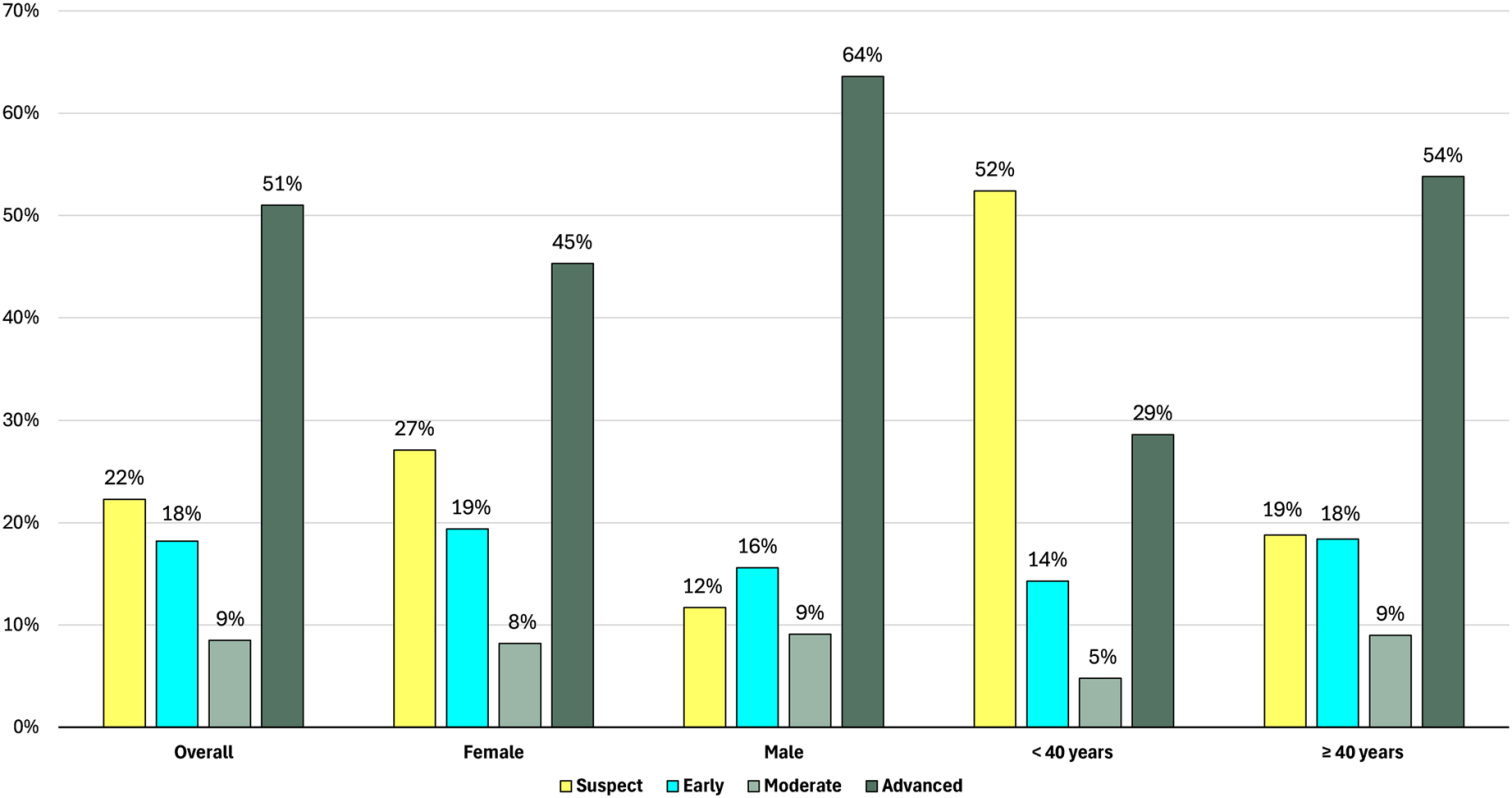
Glaucoma severity status among KPH ophthalmology patients, stratified by age and gender. *Distribution of glaucoma severity among included eyes. Distributions are shown for age, sex, and glaucoma severity at presentation among the 324 included patients*

**Fig. 3:**
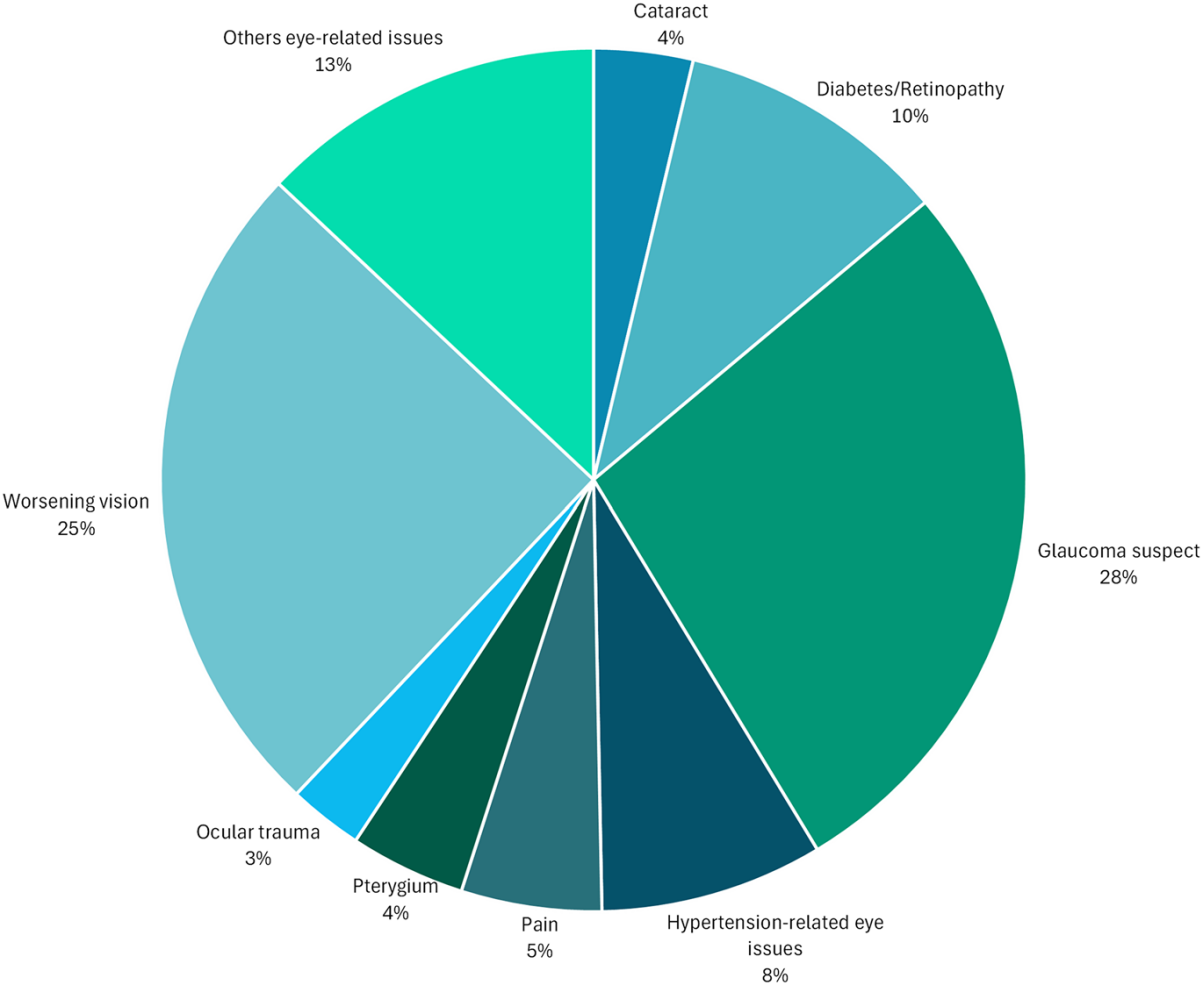
Distribution of referral reason at the patient intake. *The pie chart shows the proportion of participants’ referral reasons to the KPH ophthalmology clinic. slice sizes represent the percentage contribution of each category to the total study sample*

Charts from 324 self-identified African Caribbean patients were included in this study (Table 1). Sixty-six percent of patients were female, mean patient age was 58.3 years (± 12.3 years) and on average, men were older than women (60.86 and 56.94 years, respectively). Most charts indicated that patients referred to the KPH Ophthalmology clinic had advanced-stage glaucoma (51.0%), while 18.2% were in early stages (Fig. 2). There were significantly more advanced glaucoma cases among males (63.6%) compared to females (45.3%), and among patients ≥ 40 years old (53.8%) compared to those < 40 years (28.6%) (P < 0.05) (Fig. 2). Patient charts indicated a range of referral reasons to the KPH Ophthalmology clinic, often with multiple causes listed per patient. The most common referral reasons included suspected glaucoma (28%), worsening vision (25%), diabetic retinopathy (10%), hypertension related eye concerns (8%), and ocular trauma (3%) (Fig. 3).

**Table 1:** Demographic characteristics of Jamaican patients at KPH Ophthalmology clinic

Variable	Frequency (N = 324)	Percentage (%)
Sex		
Female	214	66.0
Male	110	34.0
*Age, Mean (SD)*	58.3 (12.3)	
18–29	6	1.9
30–39	18	5.6
40–49	47	14.6
50–59	100	31.2
60–69	87	27.1
70–79	55	17.1
80 +	8	2.5
*Age*		
< 40	24	7.5
40 +	297	92.5

SD, standard deviation

### Comorbidities and risk factors

Systemic and ocular comorbidities were common in this cohort. Cataract history was common (74.4%), whereas only 12.0% of patients had undergone cataract surgery. Ocular trauma was reported in 26.5% of charts, more frequently among males (32.7%) than females (23.4%) (Table 2). Reported trauma histories were heterogeneous and often remote and were not considered the primary etiology of glaucoma diagnosis in this cohort. Hypertension and diabetes were reported in 80.3% and 38.8% of patients, respectively (Table 3). Common symptoms at presentation included blurry vision (51.5%), floaters (21.3%), foggy vision (16.0%), excessive tearing (16.7%), and foreign body sensation (17.6%) (Table 4). Family history of glaucoma and blindness were reported by 30.9% and 19.8% of patients, respectively (Table 3). Self-reported adherence to glaucoma medications was low, with only 17.3% indicating regular use. Full details of comorbidities, ocular history, and symptom reports are summarized in Table 2. More granular trauma and symptom data are available in Online Resource 1.

**Table 2:** Self-reported and clinical ocular history of study population

Variable	Total (N = 324) n (%)	Sex category			Age category		
		Female *(n* = *214)* n (%)	Male *(n* = *110)* n (%)	*P*-value	< 40 years *(n* = *24)* n (%)	40 + years *(n* = *297)* n (%)	*P*-value
Compliant with glaucoma medication							
No	101 (31.2)	63 (29.4)	38 (34.5)	0.52	8 (33.3)	92 (31.0)	0.83
Yes	56 (17.3)	36 (16.8)	20 (18.2)		5 (20.8)	51 (17.2)	
Unknown	167 (51.5)	115 (53.7)	52 (47.3)		11 (45.8)	154 (51.9)	
History of cataracts							
No	73 (22.5)	51 (23.8)	22 (20.0)	0.17	20 (83.3)	52 (17.5)	< 0.001
Yes	241 (74.4)	159 (74.3)	82 (74.5)		4 (16.7)	235 (79.1)	
Unknown	10 (3.1)	4 (1.9)	6 (5.5)		0 (0.0)	10 (3.4)	
History of cataracts surgery							
No	282 (87.0)	190 (88.8)	92 (83.6)	0.11	23 (95.8)	256 (86.2)	0.40
Yes	39 (12.0)	21 (9.8)	18 (16.4)		1 (4.2)	38 (12.8)	
Unknown	3 (0.9)	3 (1.4)	0 (0.0)		0 (0.0)	3 (1.0)	
History of selective laser trabeculoplasty							
No	289 (89.2)	192 (89.7)	97 (88.2)	0.66	22 (91.7)	264 (88.9)	0.89
Yes	34 (10.5)	21 (9.8)	13 (11.8)		2 (8.3)	32 (10.8)	
Unknown	1 (0.3)	1 (0.5)	0 (0.0)		0 (0.0)	1 (0.3)	
History of laser iridectomy							
No	320 (98.8)	211 (98.6)	109 (99.1)	0.77	24 (100.0)	293 (98.7)	0.85
Yes	3 (0.9)	2 (0.9)	1 (0.9)		0 (0.0)	3 (1.0)	
Unknown	1 (0.3)	1 (0.5)	0 (0.0)		0 (0.0)	1 (0.3)	
History of other eye surgery							
No	282 (87.0)	180 (84.1)	102 (92.7)	0.08	21 (87.5)	258 (86.9)	0.96
Yes	41 (12.7)	33 (15.4)	8 (7.3)		3 (12.5)	38 (12.8)	
Unknown	1 (0.3)	1 (0.5)	0 (0.0)		0 (0.0)	1 (0.3)	
History of ocular trauma							
No	205 (63.3)	141 (65.9)	64 (58.2)	0.19	15 (62.5)	188 (63.3)	0.94
Yes	86 (26.5)	50 (23.4)	36 (32.7)		7 (29.2)	79 (26.6)	
Unknown	33 (10.2)	23 (10.7)	10 (9.1)		2 (8.3)	30 (10.1)

**Table 3:** Summary of relevant clinical and patient history variables

Variable	Total (N = 324) n (%)	Sex category			Age category		
		Female *(n* = *214)* n (%)	Male *(n* = *110)* n (%)	*P*-value	< 40 years *(n* = *24)* n (%)	40 + years *(n* = *297)* n (%)	*P*-value
Family clinical history							
*Family history of glaucoma*							
No	162 (50.0)	102 (47.7)	60 (54.5)	0.12	12 (50.0)	149 (50.2)	0.60
Yes	100 (30.9)	74 (34.6)	26 (23.6)		9 (37.5)	89 (30.0)	
Unknown	62 (19.1)	38 (17.8)	24 (21.8)		3 (12.5)	59 (19.9)	
*Family history of blindness*							
No	221 (68.2)	145 (67.8)	76 (69.1)	0.96	17 (70.8)	202 (68.0)	0.84
Yes	64 (19.8)	43 (20.1)	21 (19.1)		5 (20.8)	58 (19.5)	
Unknown	39 (12.0)	26 (12.1)	13 (11.8)		2 (8.3)	37 (12.5)	
Lifestyle characteristics							
*Ever smoked*							
No	216 (66.7)	155 (72.4)	61 (55.5)	< 0.001	14 (58.3)	199 (67.0)	0.53
Yes	43 (13.3)	14 (6.5)	29 (26.4)		3 (12.5)	40 (13.5)	
Unknown	65 (20.1)	45 (21.0)	20 (18.2)		7 (29.2)	58 (19.5)	
*Ever drank alcohol*							
No	208 (64.2)	144 (67.3)	64 (58.2)	0.01	14 (58.3)	191 (64.3)	0.54
Yes	50 (15.4)	24 (11.2)	26 (23.6)		3 (12.5)	47 (15.8)	
Unknown	66 (20.4)	46 (21.5)	20 (18.2)		7 (29.2)	59 (19.9)	
Clinical characteristics							
*Hypertension*							
No	60 (18.7)	40 (18.8)	20 (18.5)	0.95	13 (54.2)	46 (15.6)	< 0.001
Yes	261 (80.3)	173 (81.2)	88 (81.5)		11 (45.8)	248 (84.4)	
*Diabetes*							
No	194 (61.2)	121 (57.9)	73 (67.6)	0.09	16 (69.6)	176 (60.5)	0.39
Yes	123 (38.8)	88 (42.1)	35 (32.4)		7 (30.4)	115 (39.5)

**Table 4:** Ocular symptoms at intake

Variable	Total (N = 324) n (%)	Sex category			Age category		
		Female *(n* = *214)* n (%)	Male *(n* = *110)* n (%)	*P*-value	< 40 years *(n* = *24)* n (%)	40 + years *(n* = *297)* n (%)	*P*-value
Flashes							
No	282 (87.0)	184 (86.0)	98 (89.1)	0.40	19 (79.2)	260 (87.5)	0.17
Yes	39 (12.0)	27 (12.6)	12 (10.9)		4 (16.7)	35 (11.8)	
Unknown	3 (0.9)	3 (1.4)	0 (0.0)		1 (4.2)	2 (0.7)	
Floaters							
No	251 (77.5)	171 (79.9)	80 (72.7)	0.07	19 (79.2)	230 (77.4)	0.36
Yes	69 (21.3)	39 (18.2)	30 (27.3)		4 (16.7)	64 (21.5)	
Unknown	4 (1.2)	4 (1.9)	0 (0.0)		1 (4.2)	3 (1.0)	
Blurry Vision							
No	153 (47.2)	113 (52.8)	40 (36.4)	0.004	14 (58.3)	137 (46.1)	0.17
Yes	167 (51.5)	97 (45.3)	70 (63.6)		9 (37.5)	157 (52.9)	
Unknown	4 (1.2)	4 (1.9)	0 (0.0)		1 (4.2)	3 (1.0)	
Foggy Vision							
No	270 (83.3)	180 (84.1)	90 (81.8)	0.46	21 (87.5)	248 (83.5)	0.84
Yes	52 (16.0)	32 (15.0)	20 (18.2)		3 (12.5)	47 (15.8)	
Unknown	2 (0.6)	2 (0.9)	0 (0.0)		0 (0.0)	2 (0.7)	
Redness							
No	274 (84.6)	182 (85.0)	92 (83.6)	0.38	22 (91.7)	250 (84.2)	0.09
Yes	47 (14.5)	29 (13.6)	18 (16.4)		1 (4.2)	45 (15.2)	
Unknown	3 (0.9)	3 (1.4)	0 (0.0)		1 (4.2)	2 (0.7)	
Photophobia							
No	294 (90.7)	198 (92.5)	96 (87.3)	0.06	23 (95.8)	268 (90.2)	0.08
Yes	27 (8.3)	13 (6.1)	14 (12.7)		0 (0.0)	27 (9.1)	
Unknown	3 (0.9)	3 (1.4)	0 (0.0)		1 (4.2)	2 (0.7)	
Itchiness							
No	287 (88.6)	185 (86.4)	102 (92.7)	0.17	22 (91.7)	262 (88.2)	0.14
Yes	34 (10.5)	26 (12.1)	8 (7.3)		1 (4.2)	33 (11.1)	
Unknown	3 (0.9)	3 (1.4)	0 (0.0)		1 (4.2)	2 (0.7)	
Pain							
No	240 (74.1)	155 (72.4)	85 (77.3)	0.34	17 (70.8)	221 (74.4)	0.23
Yes	81 (25.0)	56 (26.2)	25 (22.7)		6 (25.0)	74 (24.9)	
Unknown	3 (0.9)	3 (1.4)	0 (0.0)		1 (4.2)	2 (0.7)	
Dryness							
No	303 (93.5)	195 (91.1)	108 (98.2)	0.04	22 (91.7)	278 (93.6)	0.22
Yes	18 (5.6)	16 (7.5)	2 (1.8)		1 (4.2)	17 (5.7)	
Unknown	3 (0.9)	3 (1.4)	0 (0.0)		1 (4.2)	2 (0.7)	
Epiphora							
No	267 (82.4)	176 (82.2)	91 (82.7)	0.45	20 (83.3)	246 (82.8)	0.21
Yes	54 (16.7)	35 (16.4)	19 (17.3)		3 (12.5)	49 (16.5)	
Unknown	3 (0.9)	3 (1.4)	0 (0.0)		1 (4.2)	2 (0.7)	
Foreign Body Sensation							
No	264 (81.5)	178 (83.2)	86 (78.2)	0.19	18 (75.0)	243 (81.8)	0.21
Yes	57 (17.6)	33 (15.4)	24 (21.8)		5 (20.8)	52 (17.5)	
Unknown	3 (0.9)	3 (1.4)	0 (0.0)		1 (4.2)	2 (0.7)	
Swelling							
No	319 (98.5)	211 (98.6)	108 (98.2)	0.77	22 (91.7)	294 (99.0)	0.01
Yes	5 (1.5)	3 (1.4)	2 (1.8)		2 (8.3)	3 (1.0)	

### Demographic differences in risk factors

The prevalence of selected comorbidities, lifestyle factors, and ocular history differed by sex and age group. A significantly higher proportion of males reported smoking (26.4%) and alcohol use (23.6%) compared to females (Table 3) (P < 0.05). Hypertension was more prevalent among patients aged ≥ 40 (84.4%) compared to those < 40 (45.8%; P < 0.001). and cataract history was also more common in the older group (79.1% vs. 16.7%; P < 0.001) (Table 2). Males were more likely to report a history of ocular trauma (32.7% vs. 23.4%) (Table 2) and blurred vision (63.6% vs. 45.3%) (Table 4), while females more frequently reported symptoms like eye dryness (P = 0.04). These patterns suggest that vascular, ocular, and behavioral risk factors may differ by sex and age in this population (Table 2; Online Resource 1).

### Optic nerve head (ONH) parameters

OCT-derived optic nerve head and retinal nerve fiber layer parameters demonstrated structural changes consistent with glaucomatous damage in this cohort. The average RNFL thickness was 75.2 ± 14.9 μm, with average RA of 0.96 ± 0.30 mm^2^ and DA of 2.17 ± 0.43 mm^2^ (Table 5). Median cup volume was 0.52 mm^3^ (IQR: 0.34–0.73), and RNFL symmetry was 69.0% (IQR: 45.5–81.0). Across age groups, patients < 45 years had higher signal strength (8.14 ± 0.96 vs. 7.84 ± 1.04) and thicker RNFL (77.54 ± 15.3 μm vs. 74.9 ± 14.8 μm), although differences in DA, CDR, and VCDR by age were not statistically significant. RNFL SNIT quadrant averages were 90.4 ± 21.9 μm (S), 65.4 ± 11.4 μm (N), 90.7 ± 25.4 μm (I), and 54.0 ± 12.5 μm (T) (Table 6). No significant SNIT quadrant differences were observed by sex or age group. ONH measures were comparable to prior studies among populations of African and European ancestry (Table 7); OCT findings from most patients in this study were consistent with advanced POAG disease. The RNFL thickness distribution across sectors (Superior, Nasal, Inferior, Temporal) in both eyes showed progressively thinner RNFLs as glaucoma severity increased (Online Resource 2). The Inferior and Temporal sectors showed greater thickness differences between severity groups (Online Resource 3; Online Resource 4).

**Table 5:** Age-stratified OCT parameters at initial exam

Variable	Total *(n* = *324) Mean (SD)*	Sex Category			Age Category		
		Female *(n* = *214) Mean (SD)*	Male *(n* = *110) Mean (SD)*	*P*-value	< 40 years *(n* = *24) Mean (SD*)	40 + years *(n* = *297) Mean (SD)*	*P*-value
Signal strength							
Bilateral	7.87 (1.04)	7.92 (1.04)	7.78 (1.04)	0.26	8.14 (0.96)	7.84 (1.04)	0.15
Right	7.85 (1.20)	7.87 (1.21)	7.81 (1.20)	0.71	7.91 (1.38)	7.84 (1.19)	0.80
Left	7.95 (1.15)	8.01 (1.15)	7.81 (1.15)	0.15	8.45 (1.01)	7.89 (1.15)	0.02
Average RNFL thickness (μm)							
Bilateral	75.20 (14.90)	75.15 (14.76)	75.23 (15.30)	0.97	77.54 (15.28)	74.87 (14.79)	0.42
Right	76.30 (17.10)	75.88 (16.93)	77.14 (17.40)	0.55	79.65 (15.96)	75.90 (17.17)	0.29
Left	75.30 (17.7)	75.87 (17.74)	74.07 (17.79)	0.41	76.77 (17.34)	75.02 (17.16)	0.65
Rim area (mm^2^)							
Bilateral	0.96 (0.30)	0.97 (0.32)	0.93 (0.27)	0.18	0.90 (0.34)	0.96 (0.30)	0.43
Right	0.97 (0.33)	0.99 (0.34)	0.96 (0.30)	0.50	0.98 (0.35)	0.97 (0.33)	0.96
Left	0.95 (0.34)	0.97 (0.34)	0.90 (0.32)	0.07	0.86 (0.36)	0.95 (0.33)	0.25
Disc area (mm^2^)							
Bilateral	2.17 (0.43)	2.17 (0.41)	2.15 (0.46)	0.61	2.15 (0.33)	2.16 (0.43)	0.91
Right	2.17 (0.44)	2.19 (0.41)	2.14 (0.50)	0.36	2.21 (0.35)	2.16 (0.45)	0.58
Left	2.17 (0.45)	2.18 (0.45)	2.15 (0.46)	0.54	2.10 (0.36)	2.17 (0.46)	0.35
Disc area average cup-to-disc ratio							
Bilateral	0.73 (0.12)	0.72 (0.13)	0.74 (0.10)	0.31	0.74 (0.12)	0.73 (0.12)	0.53
Right	0.73 (0.12)	0.72 (0.13)	0.73 (0.10)	0.72	0.72 (0.13)	0.73 (0.12)	0.54
Left	0.73 (0.13)	0.72 (0.13)	0.74 (0.13)	0.24	0.75 (0.13)	0.73 (0.13)	0.46
Vertical cup-to-disc ratio							
Bilateral	0.71 (0.12)	0.70 (0.13)	0.72 (0.11)	0.24	0.72 (0.13)	0.71 (0.12)	0.69
Right	0.70 (0.13)	0.70 (0.14)	0.71 (0.12)	0.44	0.69 (0.13)	0.70 (0.13)	0.76
Left	0.71 (0.14)	0.70 (0.14)	0.72 (0.15)	0.30	0.72 (0.13)	0.71 (0.14)	0.65
Cup volume (mm^3^)							
Bilateral *median (IQR)*	0.52 (0.34–0.73)	0.51 (0.3–0.71)	0.52 (0.36–0.74)	0.44	0.56 (0.32–0.91)	0.52 (0.34–0.71)	0.38
Right, *median (IQR)*	0.49 (0.32–0.73)	0.50 (0.32–0.73)	0.49 (0.32–0.72)	0.69	0.50 (0.32–0.83)	0.49 (0.32–0.71)	0.74
Left							
*median (IQR)*	0.53 (0.30–0.74)	0.51 (0.30–0.73)	0.56 (0.34–0.81)	0.31	0.59 (0.30–0.92)	0.53 (0.30–0.74)	0.37
RNFL Symmetry							
*Median (IQR)*	69.0 (45.5–81.0)	69.0 (46.2–83.8)	67.0 (43.0–78.0)	0.44	72.0 (48.0–83.0)	69.0 (46.0–81.0)	0.58

OCT, ocular coherence tomography; IQR, interquartile range; RNFL, retinal nerve fiber layer

**Table 6:** Retinal nerve fiber layer (RNFL) thickness by quadrant at first exam

Variable	Total N = 324 *Mean (SD)*	Sex Category			Age Category		
		Female *n* = *214 Mean (SD)*	Male *n* = *110 Mean (SD)*	*P*-value	< 40 years n = 24 *Mean (SD)*	40 + years n = 297 *Mean (SD)*	*P*-value
Superior							
Bilateral	90.37 (21.89)	90.28 (21.59)	90.53 (22.55)	0.93	92.71 (24.47)	90.11 (21.71)	0.62
Right	90.42 (26.34)	89.91 (26.43)	91.43 (26.24)	0.64	93.39 (28.02)	89.94 (26.21)	0.57
Left	91.94 (26.18)	92.53 (25.34)	90.73 (27.88)	0.59	93.45 (25.59)	91.92 (25.74)	0.79
Nasal							
Bilateral	65.40 (11.40)	64.90 (11.69)	66.37 (10.98)	0.27	69.23 (11.81)	64.93 (11.09)	0.09
Right	66.30 (13.20)	65.64 (13.46)	67.61 (12.79)	0.22	72.35 (13.14)	65.80 (13.21)	0.03
Left	65.29 (14.51)	65.16 (15.83)	65.55 (11.52)	0.81	66.41 (12.72)	64.86 (13.57)	0.59
Inferior							
Bilateral	90.70 (25.36)	90.54 (25.33)	91.02 (25.54)	0.87	93.06 (26.69)	90.47 (25.37)	0.65
Right	93.04 (29.42)	92.55 (29.53)	94.01 (29.32)	0.69	97.70 (26.95)	92.42 (29.64)	0.38
Left	89.86 (29.27)	90.48 (28.82)	88.61 (30.23)	0.61	91.50 (32.20)	89.87 (28.83)	0.82
Temporal							
Bilateral	54.00 (12.54)	54.55 (13.32)	52.92 (10.87)	0.24	55.65 (12.95)	53.62 (11.99)	0.47
Right	53.89 (17.57)	55.17 (19.70)	51.32 (11.96)	0.04	55.73 (13.10)	53.31 (16.18)	0.42
Left	55.11 (12.82)	54.92 (12.60)	55.48 (13.28)	0.73	56.09 (14.78)	54.97 (12.71)	0.73

**Table 7:** Summary of mean optic nerve parameters reported in the Jamaica eye study (early, moderate, advanced glaucoma) and prior published studies

Study	Year	Population	Sample Size	Diagnostic group	Eye selection	VCDR	DA (mm^2^)	RNFL thickness (µm)
Jamaica Eye Study	2025	African Caribbean	324	Glaucoma	Both Eyes	0.7 ± 0.1	2.17 ± 0.4	75.2 ± 14.9
Leske et al. [32]	1994	African Caribbean	309	OAG	Either Eye	0.8 ± 0.1	–	–
Seider et al.[47]	2009	European Descent	215	Glaucoma	Both Eyes	–	2.15 ± 0.6	–
		African American	67			–	2.55 ± 0.5	
Sample et al.[48]	2009	African Descent	185	Glaucoma	Either Eye	0.80 ± 0.1	–	–
		European Descent	184			0.82 ± 0.1	–	–
Tatham et al.[49]	2013	Mixed	53	Glaucoma Suspect	Either Eye	0.65 ± 0.1	1.95 ± 0.4	84.0 ± 10.2
Bokman et al.[50]	2014	African Caribbean	191	Glaucoma Suspect	Either Eye	0.6 ± 0.2		
Gracitelli et al.[51]	2018	African Descent	171	POAG	Both Eyes	–	–	78.9 ± 17.5
		European Descent	173			–	–	74.1 ± 15.5

^*^Comparison of mean optic nerve parameter measurements between African Caribbean, African American, European Descent, and White individuals in studies comparing racial or ethnic variations in glaucomatous eyes;

VCDR, vertical cup-to-disc ratio; DA, disc area; RNFL, retinal nerve fiber layer; POAG, primary open angle glaucoma; OAG, open angle glaucoma; OCT, ocular coherence tomography; FDT, frequency doubling technology

### Intraocular pressure

IOP measurements were available using both NCT and GAT. Mean IOP measured by NCT was 20.4 ± 6.5 mmHg (OD) and 21.3 ± 7.3 mmHg (OS), while GAT readings averaged 22.0 ± 6.7 mmHg (OD) and 22.6 ± 7.6 mmHg (OS) (Table 8). Male patients had a higher right-eye GAT IOP values compared to females (*P* = 0.05), consistent with more advanced disease at presentation. Higher IOP among males may reflect delayed care-seeking or more severe disease at presentation.

**Table 8:** Clinical Exam Parameters Stratified by Subgroup

		Sex category			Age category		
Variable	Total (*n* = 324) *Mean (SD)*						
		Female *(n* = *214) Mean (SD)*	Male *(n* = *110) Mean (SD)*	*P*-value	18–40 years *(n* = *24) Mean (SD)*	40 + years (n = 297) *Mean (SD)*	*P*-value
OD NCT IOP (mmHg)	20.40 ± 6.45	20.35 ± 6.11	20.50 ± 7.24	0.87	19.06 ± 3.87	20.54 ± 6.65	0.16
OS NCT IOP (mmHg)	21.30 ± 7.32	21.00 ± 6.30	21.93 ± 9.16	0.41	21.28 ± 8.28	21.31 ± 7.28	0.99
OD GAT IOP (mmHg)	22.0 ± 6.72	20.96 ± 5.49	23.92 ± 8.41	0.05	19.55 ± 5.34	22.21 ± 6.8 1	0.19
OS GAT IOP (mmHg)	22.60 ± 7.59	21.94 ± 5.96	23.78 ± 10.01	0.29	19.33 ± 7.37	22.89 ± 7.58	0.20
OD Visual Acuity *(LogMAR) median (IQR)*	0.30 (0.10, 0.60)	3.0 (0.1, 0.54)	0.3 (0.1, 1.0)	0.35	0.0 (0.0, 0.1)	0.3 (0.10, 0.60)	< 0.001
OS Visual Acuity *(LogMAR) median (IQR)*	0.30 (0.10, 0.54)	0.18 (0.1, 0.54)	0.40 (0.1, 0.60)	0.07	0.0 (0.0, 0.18)	0.3 (0.1, 0.6)	0.001

OD, oculus dexter; OS, oculus sinister; NCT, non-contact tonometry; GAT, goldmann applanation tonometry; IOP, intraocular pressure; IQR, interquartile range

### Visual acuity

VA at presentation was assessed using LogMAR measurements and summarized by each eye. Median LogMAR visual acuity was 0.3 (IQR: 0.1–0.6) in both OD and OS. VA was significantly different by age group, but no significant differences in VA were observed by sex (Table 8). Despite advanced disease, central VA was moderately preserved in most patients at the time of evaluation.

### Visual field testing

VF testing was available for a subset of patients and was inconsistently documented across the cohort, limiting assessment of longitudinal functional change. Among patients with available VF data (Table 9), 76.5% had a single test; 18.6% had two, and only 2.8% had three or more, limiting longitudinal evaluation. In the right eye, the median visual field index (VFI) was 89.0% (IQR: 62.5–97.0%), mean deviation (MD) was –6.7 dB (IQR: −14.1 to −3.9), and pattern standard deviation (PSD) was 3.7 dB (IQR: 2.0–6.7). Males demonstrated worse VF performance than females, with median MD of −7.8 dB versus −6.1 dB. Median VFI was lower in males (84.5%) than females (90.0%). PSD was higher in males but not significant (P = 0.14), potentially indicating more localized visual loss. Despite missing VF data in 23% of charts, these patients were retained based on other diagnostic indicators, such as optic nerve appearance, documented CDR and baseline IOP. VF data confirmed moderate functional loss and highlighted disparities in longitudinal monitoring capacity.

**Table 9:** Visual field index of the left and right eyes of screened participant in KPH

		Sex category			Age category		
Variable	Total *(N* = *324) median (IQR)*	Female *(n* = *214) median (IQR)*	Male *(n* = *110) median (IQR)*	*P*-value	18–40 years *(n* = *24) median (IQR)*	40 + years (n = 297) *median (IQR)*	*P*-value
Right eyes							
Visual field index (%)*)*	89.0 (62.5, 97.0)	90.0 (70.0, 97.0)	84.5 (40.3, 96.0)	0.18	96.0 (88.0, 98.5)	88.0 (56.0, 96.0)	0.04
Mean deviation (dB)	−6.65 (−14.1, −3.85)	−6.11 (−12.70, −3.76)	−7.82 (−16.5, −5.19)	0.20	−2.99 (−6.41, −1.19)	−7.15 (−15.6, −4.41)	0.001
Pattern standard deviation (dB)	3.72 (2.01, 6.67)	3.26 (2.01, 6.38)	5.18 (2.06, 7.24)	0.14	1.96 (1.67, 4.81)	4.07 (2.09, 6.83)	0.04
Left eyes							
Visual field index (%)	89.0 (58.0, 96.0)	90.0 (57.0, 96.0)	86.0 (58.0, 93.0)	0.38	94.0 (93.0, 99.0)	88.0 (54.0, 95.0)	0.01
Mean deviation (dB)	−7.17 (−16.8, 4.06)	−7.0 (−16.40, −3.94)	−9.17 (−22.4, −4.48)	0.28	−2.12 (−4.53, −1.64)	−7.76 (−17.5, −4.75)	< 0.001
Pattern standard deviation (dB)	3.94 (2.06, 6.90)	3.61 (1.97, 6.54)	4.96 (2.29, 7.66)	0.16	2.07 (1.46, 3.65)	4.42 (2.09, 7.30)	0.003
Bilateral eyes							
Visual field index (%)	86.5 (53.2, 94.8)	89.8 (54.8, 95.5)	79.5 (51.0, 92.5)	0.25	97.0 (94.60 99.0)	83.8 (50.8, 93.1)	< 0.001
Mean deviation (dB)	−7.72 (−16.6, −4.53)	−6.62 (−16.6, −4.53)	−9.75 (−16.6, −4.68)	0.43	−3.31 (−5.42, −1.86)	−8.11 (−16.6, −4.72)	0.01
Pattern standard deviation (dB)	4.18 (2.36, 6.33)	4.09 (2.28, 6.19)	4.61 (2.67, 6.79)	0.28	2.36 (1.65, 4.30)	4.37 (2.5, 6.47)	0.01
Visual field test							
One	248 (76.5)	175 (81.8)	73 (66.4)	0.002	19 (79.2)	226 (76.1)	0.73
Two	46 (18.6)	42(24.7)	4(5.2)	< 0.001	8 (38.1)	37 (16.6)	0.02
Three	7 (2.8)	7 (4.1)	0 (0.0)	0.07	2 (9.5)	5 (2.2)	0.06
More than 3	2 (0.8)	2 (1.2)	0 (0.0)	0.34	1 (4.8)	1 (0.4)	0.04

IQR, interquartile range

## Discussion

### Key findings and clinical implications

This study provides the first large-scale characterization of clinical, demographic, and structural features among glaucoma patients receiving care at a public ophthalmology clinic in Jamaica and reveals a striking burden of advanced disease presentation. More than half of patients presented with documented visual impairment at their first encounter, characterized by peripheral visual field loss (as evidenced by a peripheral scotoma). 17% self-reported adherence with glaucoma medications, emphasizing substantial challenges in early detection and disease self-management. Mean CDR, RNFL thinning, and IOP measurements reflected predominantly late-stage disease. By anchoring structural OCT and clinical measures in an African-Caribbean cohort, these findings provide the foundational evidence needed to inform targeted interventions in glaucoma detection, monitoring, and treatment. Systemic comorbid conditions, especially hypertension and diabetes, were more common than the national estimates [29, 52], aligning with prior literature on vascular contributions to glaucoma pathogenesis in populations of African descent[53].

### Health system and diagnostic barriers to glaucoma care

Although Jamaica’s public eye care infrastructure is concentrated in the metropolitan parishes of Kingston, St. Andrew, and St. James, with over 75% of eyecare services located in these areas, only 30.9% of the Island’s population resides in these areas [54, 55]. This geographic mismatch highlights significant disparities in access to ophthalmic services across the island [55–57]. Most patients in our study were seen at KPH approximately twice per year, with a mean interval of 202 days between follow-up visits. This frequency (two annual exams) aligns with international guidelines for stable glaucoma management [58]; however, patients with unstable disease, including those with fluctuating IOP, VF changes, or ON progression—as well as those with multiple risk factors—require more frequent follow up. Unfortunately, this more intensive follow-up is often not consistently achievable within the public hospital system due to persistent barriers such as equipment shortages, limited clinic capacity, and fragmented referral networks, particularly in rural and underserved parishes [55, 59].

Glaucoma monitoring at KPH Ophthalmology clinic reveals critical diagnostic limitations shaped by equipment constraints, paper-based records, and broader systemic inequities. Although most patients in this study underwent at least one VF test, few received three or more during their care at KPH. The average interval between the first and second VF tests was 1,262 days, and the interval between the second and third tests was even longer (2,202 days), substantially limiting the ability to detect meaningful changes in VF defects over time. Delayed recognition of VF worsening is clinically consequential, as functional deterioration often prompts escalation of therapy, including intensification of medical management or consideration of surgical intervention, particularly when interpreted alongside structural findings and IOP measurements. A key driver of these prolonged testing intervals was extended distribution of VF services: the VF machine at KPH was unavailable for nearly two years, requiring patients to obtain testing at private clinics at personal cost (approximately $32 USD) and return with results for continued care—steps that likely introduced additional barriers related to affordability, transportation, and follow-through. These disruptions, alongside intermittent equipment malfunction, staffing shortages, healthcare worker strikes, and disruptions during the *COVID-19* pandemic, reflect broader system-level constraints affecting longitudinal functional monitoring.

Beyond structural and functional testing limitations, physiologic factors that influence glaucoma risk stratification and IOP interpretation also warrant consideration. Central corneal thickness (CCT) is an important modifier of IOP measurement and can impact risk assessment, particularly in populations of African descent [60]. Prior studies have demonstrated that individuals with African genetic ancestry tend to have thinner mean CCT compared with populations of European ancestry, with higher proportions of African genetic ancestry associated with even thinner corneas [60, 61]. Thinner CCT can lead to underestimation of true IOP when measured by GAT, potentially obscuring disease severity and delaying treatment escalation [62]. This limitation is especially relevant in the Jamaican context, where patients often present with advanced glaucomatous damage and accurate IOP interpretation is critical for setting appropriate target pressures. While CCT refines IOP interpretation and supports individualized management, glaucoma diagnosis remains based on structural ON changes and functional VF loss rather than IOP alone. At KPH, CCT measurement has been available since 2019 following the donation of pachymetry and specular microscopy equipment, representing an important enhancement in diagnostic capacity and longitudinal risk stratification.

### Sex differences at presentation

This cohort reflects patients who successfully accessed tertiary ophthalmic care. Men presented with higher IOP (NCT and GAT) a finding that may reflect delayed care-seeking behavior, reduced health awareness, or lower adherence to treatment, consistent with patterns previously reported among Jamaican men [28, 63]. Additionally, a higher proportion of men (32.7%) reported a history of ocular trauma compared to women (23.4%); while this difference was not statistically significant, it may suggest that lifestyle or occupational exposures contribute to more advanced disease at presentation [33, 35].These findings align with prior evidence suggesting gender-based disparities in glaucoma care and disease severity [64].

### Reliance on OCT for glaucoma monitoring: structural insight and normative bias

In the setting of infrequent and unreliable visual field testing, access to OCT equipment is central for structural assessment, disease staging, and clinical decision-making for treatment and management for glaucoma patients at KPH. OCT is a routine component of glaucoma care, with pharmacological dilation and lubricating drops administered as needed to optimize scan quality. In the present study, only charts with high-quality OCT scans (signal strength ≥ 6) were included in the final analysis. Several records (n = 868 of the 1,489) were excluded due to poor-quality scans, reflecting broader infrastructure and training/user challenges that affect consistent imaging access. Despite these constraints, OCT-derived structural parameters, including RNFL thinning and enlarged cup-to-disc-ratios, provided critical insight into disease severity and supported clinical staging, often in the absence of reliable longitudinal VF data.

Interpretation of these findings, however, must be contextualized within a key diagnostic limitation: most commercial OCT platforms rely on normative reference databases that underrepresent individuals of African ancestry, including African Caribbean populations [4, 18, 65]. Prior studies have demonstrated systematic differences in ONH morphology, DA, and RNFL thickness between individuals of African and European ancestry, such that structurally distinct eyes evaluated against non-representative normative references may have glaucomatous damage underestimated or detected later in the disease course [4, 66, 67]. The advanced structural damage observed at presentation in this cohort therefore likely reflects both true late-stage disease at referral and delayed structural flagging due to normative mismatch.

These challenges are further amplified by the fact that KPH Ophthalmology clinic houses the only OCT machine currently available in Jamaica’s public hospital system, requiring patients in other regions to seek private imaging or await referral, thereby creating significant delays and access inequities in structural imaging [65]. Together, these findings underscore the urgent need to expand ancestrally and regionally representative OCT normative databases and to invest in complementary, accessible functional testing strategies to improve diagnostic accuracy, disease staging, and equitable glaucoma care across the Caribbean.

The larger mean optic disc area observed in this cohort also raises important considerations regarding the risk of pseudo glaucoma. In populations with larger physiologic discs, reliance on color-coded sector maps alone may increase the likelihood of misclassification, particularly when normative reference databases do not adequately reflect ancestral variation [16, 19]. Historically, such cases were more likely to be mislabeled in the absence of objective structural or functional testing. In current practice, however, patients with large discs are increasingly classified as glaucoma suspects and referred for further evaluation using OCT and VF testing [68, 69]. The availability of high-quality OCT data in this study represents a key strength, enabling more accurate differentiation between physiologic cupping and glaucomatous structural damage.

### Comparisons to prior literature

Our findings both align with and diverge from previously published studies of glaucoma in African Caribbean and other populations of African descent. Overall, disease burden and severity were consistent with those reported in population-based studies such as the Barbados Eye Study [32]. However, ONH parameters, including DA and CDR, more closely resemble European-descent populations, rather than in African American or African Caribbean cohorts from the U.S.-based African Descent and Glaucoma Evaluation Study (ADAGES)[48, 66, 70].

The observation that selected ONH parameters in this cohort more closely resemble values reported in European-descent populations should be interpreted cautiously. This finding likely reflects several intersecting factors, including genetic heterogeneity within African-descent populations, differences in disease stage and referral patterns across studies, and methodological variation in OCT acquisition, segmentation algorithms, and normative reference databases. Comparisons across studies are further influenced by whether cohorts include predominantly early versus advanced disease, as well as by platform-specific definitions of ONH metrics. Rather than suggesting convergence across populations, these findings highlight the substantial within-group variability that exists among individuals of African ancestry and reinforce the need for ancestrally and regionally representative normative data to support accurate interpretation.

Glaucoma burden in Jamaica has historically been underrepresented in national surveillance. A 2012 national survey found that 12% of Jamaicans over age 60 reported a prior glaucoma diagnosis, with high comorbidity rates: 61.4% had hypertension and 26.2% had diabetes [29]. However, more recent studies suggest this may be an underestimate. For example, a clinic-based study reported a glaucoma prevalence of 29.6%, raising concerns about delayed diagnosis and the unrecognized scope of disease burden [55]. Similarly, an assessment of vision-related quality of life among glaucoma patients at KPH and found that lower visual acuity, longer disease duration, and higher medication burden were associated with poorer quality of life, mirroring findings from the Barbados Eye Study and other global cohorts [28, 32].

Medication adherence remains a persistent challenge in this context. Mowatt et al. reported that only 22% of patients in a Jamaican hospital setting demonstrated adequate understanding of their glaucoma diagnosis, with approximately 50% fully adherent to prescribed therapy, more than double the adherence rate observed in our cohort (17.3%) [71]. Taken together, these findings highlight critical gaps in patient education, treatment adherence, and continuity of care, alongside broader structural limitations within Jamaica’s eye care system. These include shortages of ophthalmology clinics, uneven geographic distribution of services, limited access to diagnostic technologies such as OCT and VF machines and fragmented referral pathways that delay early detection and timely intervention [65, 72]. Qualitative notes within patient charts frequently cited, forgetfulness, perceived lack of benefit, medication side effects, and financial strain as contributors to non-compliance, underscoring the multifactorial nature of treatment challenges in this cohort.

### Public health implications

Together, these clinical, educational, and systemic barriers underscore the need for coordinated public health strategies to improve access, strengthen care continuity, and promote better glaucoma outcomes across Jamaica. The predominance of moderate to advanced disease at presentation in this cohort highlights persistent gaps in early detection, patient awareness, and timely access to eye care services. Because glaucoma is largely asymptomatic in its early stages routine screening and prompt diagnosis are essential to preserving vision [73]. However, in many low- and middle-income settings, patients often seek care only after experiencing noticeable vision loss, substantially limiting the effectiveness of available treatments [26, 59, 74]. Similar patterns have been reported across the Caribbean and sub-Saharan Africa, frequently attributed to low disease awareness, limited access to specialist care, and fragmented referral systems [75, 76]. Targeted screening and education efforts focused on high-risk groups, including adults over age 40, individuals with a family history of glaucoma, and populations of African descent, are likely to be more cost-effective and impactful than untargeted population-wide approaches [77, 78, 79]. The high prevalence of chronic disease and ocular trauma in this cohort further supports the need for integrated, multidisciplinary eye health strategies, including alignment of glaucoma education and screening with existing non-communicable disease prevention initiatives and coordinated care across primary, secondary, and tertiary health systems. 

### Strengths and limitations

This study represents the first large-scale, clinic-based characterization of glaucoma in Jamaica, offering critical insight into disease presentation and OCT-derived structural findings within a public healthcare setting. Key strengths include a robust sample size (N = 324 patients; 619 glaucomatous eyes), systematic chart abstraction, and the integration of detailed clinical and OCT imaging data. The inclusion of both structural OCT parameters and clinical measures allowed for a comprehensive assessment of disease status and documentation of real-world care delivery within a tertiary referral center in the Caribbean. While innovative and timely, several limitations must be acknowledged. As a retrospective, hospital-based study conducted at a national tertiary referral center, this analysis captures patients who accessed specialized ophthalmic care and had documented OCT imaging. Consequently, the cohort is likely enriched for individuals with more advanced or symptomatic glaucoma, and disease severity observed in this study may overestimate the distribution of severity in the general Jamaican population. Findings therefore reflect characteristics of diagnosed and treated glaucoma in a tertiary care setting rather than the full burden of undiagnosed the broader community. This inherent selection bias aligns with the ‘50% rule’ [80], which theorizes that approximately half of glaucoma cases remain undiagnosed in the community. Future prospective, population-based studies, modeled on recruitment practices the Barbados Eye Study [32], are essential to accurately determine prevalence, risk factors, and detection gaps across Jamaica.

Additional limitations include incomplete paper-based records, particularly regarding socioeconomic data, medication adherence, and follow-up patterns; approximately 42.7% of charts lacked complete information on socioeconomic or compliance information. Variability in examiner technique, documentation standards, and equipment calibration may have introduced measurement inconsistencies, particularly in IOP and VA assessments. VF reliability was low in many charts, reflecting both equipment downtime and operational constraints during the study period (January 2018-March 2023). The absence of a control or comparison group also limits the ability to contextualize disease characteristics and imaging parameters relative to non-glaucomatous patients in this population. Although a subset of patients reported a history of ocular trauma, these cases are being evaluated separately in an ongoing secondary analysis focused on trauma-related ocular history and glaucoma presentation and were therefore not stratified or reclassified within the present POAG-focused study. Despite these challenges, this study provides a valuable foundation for improving glaucoma surveillance and care in the Caribbean. The data highlight diagnostic and structural barriers within the public health system and establish the groundwork for structured, prospective, and community-based research aimed at equitable detection, monitoring, and management of glaucoma in Jamaica.

## Conclusion

This study provides critical clinical insight into glaucoma among African Caribbean patients receiving care in Jamaica’s public health system. The high prevalence of advanced disease at presentation, coupled with substantial systemic comorbidity and low medication adherence, highlights persistent gaps in early detection and continuity of care. By characterizing patterns of glaucoma severity and structural disease features in this setting, these findings underscore how delayed presentation, limited diagnostic capacity, and health system constraints may contribute to vision loss in underserved populations. Importantly, this work establishes a foundation for improved clinical documentation, culturally responsive interventions, and the development of more inclusive diagnostic and management approaches tailored to African Caribbean patients, with relevance for other high-risk populations of African descent.

### Future directions

Improving glaucoma care in Jamaica and similar resource-constrained settings will require prospective studies with standardized documentation and longitudinal follow-up. Systematic collection of VF metrics, OCT-derived ON and RNFL parameters, IOP trends, and relevant systemic health data will enable more accurate disease staging and monitoring over time. Efforts to improve diagnostic equity should include development of ancestry-aware tools, particularly expanded and representative OCT normative databases. Community-based screening initiatives, paired with mobile or portable perimetry platforms and streamlined referral pathways, may offer scalable approaches for earlier detection. At the clinic level, low-cost interventions, such as standardized glaucoma intake forms, routine documentation of family history, and adherence tracking, could improve care continuity while strengthening data quality for future research.

Given the high proportion of patients presenting with advanced disease, surgical management remains a critical component of glaucoma care in Jamaica. While surgical outcomes were beyond the scope of this analysis, the expansion of a dedicated glaucoma subspecialty service at KPH since 2018 has increased access to procedures such as trabeculectomy, selective laser trabeculoplasty, and minimally invasive surgical techniques. Ongoing and planned secondary analyses will examine surgical sequencing, outcomes, and longitudinal disease trajectories across additional cohorts. Complementing these quantitative efforts, a parallel qualitative study led by the KPH glaucoma team is currently examining patient and provider perspectives on glaucoma self-management, barriers to medication adherence, and opportunities for community-based education. Together, these integrated research directions aim to inform earlier intervention strategies and more equitable glaucoma care delivery in Jamaica and across the Caribbean.

## Supplementary Information

Below is the link to the electronic supplementary material.

Supplementary file1 (PDF 294 kb)

## Data Availability

The data that support the findings of this study were derived from de-identified clinical records at Kingston Public Hospital. Due to ethical and institutional restrictions governing the use of hospital-based patient data, the dataset is not publicly available. De-identified data may be made available from the corresponding author upon reasonable request and with appropriate institutional approvals.
