# Supplementary material for: Clinical and demographic characteristics of glaucoma patients in Kingston, Jamaica
Source: Int Ophthalmol. 2026 Apr 29;46(1):217. doi: 10.1007/s10792-026-04020-y (PMC13128785; doi:10.1007/s10792-026-04020-y)
Supplement: Supplementary file 1 — Supplementary file1 (PDF 294 kb) [file 10792_2026_4020_MOESM1_ESM.pdf]

Supplementary Materials

Title: Clinical And Demographic Characteristics of Glaucoma Patients in Kingston, Jamaica

Corresponding Author
Jessica N. Cooke Bailey
Center for Health Disparities
Department of Pharmacology and Toxicology
Brody School of Medicine
East Carolina University
Room 6S10, 600 Moye Blvd.
Greenville, NC 27834-4354
E-mail: CookeBaileyJ23@ecu.edu
Phone: 252.744.2754

Online Resource 1. Ocular Trauma and Symptom Details by Sex

Variable	Male n (%)	Female n (%)
History of ocular trauma	55 (32.7%)	31 (23.4%)
Blurry vision	84 (63.6%)	83 (45.3%)
Floaters	28 (21.2%)	41 (21.5%)
Foggy vision	20 (15.2%)	32 (17.5%)
Excessive tearing	17 (12.9%)	37 (20.2%)
Foreign body sensation	20 (15.2%)	37 (20.2%)

Online Resource 1 provides sex-specific details of ocular trauma symptoms reported by participants during visits to the Kingston Public Hospital Ophthalmology clinic

Online Resource 2. OCT Parameter at Initial Exam and Glaucoma Severity Status					
Variable	**Suspect** *Mean (SD)*	**Early** *Mean (SD)*	**Moderate** *Mean (SD)*	**Advanced** *Mean (SD)*	P-value
**Signal Strength**					
Bilateral	8.18 (1.2)	8.10 (1.0)	8.17 (1.1)	7.73 (1.0)	0.02
Right	7.91 (1.4)	8.16 (1.3)	8.24 (1.26)	7.69 (1.11)	0.08
Left	8.46 (1.3)	8.04 (0.9)	8.20 (1.2)	7.77 (1.17)	0.003
**Average RNFL**					
Bilateral	81.3 (13.4)	82.0 (13.2)	78.4 (13.1)	70.5 (15.3)	<0.001
Right	80.56 (14.9)	83.40 (15.1)	81.33 (14.4)	71.17 (17.9)	<0.001
Left	84.41 (17.8)	80.76 (14.4)	75.25 (20.1)	71.16 (18.12)	<0.001
**Rim Area**					
Bilateral	1.10 (0.3)	1.02 (0.3)	1.10 (0.3)	0.85 (0.3)	<0.001
Right	1.10 (0.3)	1.06 (0.3)	1.13 (0.3)	0.89 (0.3)	<0.001
Left	1.10 (0.3)	1.00 (0.3)	1.05 (0.3)	0.85 (0.3)	<0.001
**Disc Area**					
Bilateral	2.18 (0.5)	2.16 (0.4)	2.31 (0.4)	2.14 (0.4)	0.42
Right	2.20 (0.4)	2.13 (0.5)	2.30 (0.5)	2.14 (0.4)	0.39
Left	2.18 (0.5)	2.18 (0.4)	2.29 (0.4)	2.15 (0.5)	0.61
**Cup-to-disc-ratio**					
Bilateral	0.68 (0.1)	0.71 (0.1)	0.71 (0.1)	0.76 (0.1)	<0.001
Right	0.68 (0.1)	0.69 (0.1)	0.69 (0.1)	0.75 (0.1)	<0.001
Left	0.68 (0.1)	0.72 (0.1)	0.72 (0.1)	0.76 (0.1)	<0.001
**Vertical Cup-to-disc-ratio**					
Bilateral	0.66 (0.1)	0.68 (0.1)	0.68 (0.1)	0.75 (0.1)	<0.001
Right	0.67 (0.1)	0.67 (0.1)	0.66 (0.1)	0.73 (0.1)	<0.001
Left	0.65 (0.1)	0.69 (0.1)	0.70 (0.1)	0.75 (0.1)	<0.001
**Cup volume**					
Bilateral	0.45 (0.29, 0.64)	0.43 (0.28, 0.63)	0.39 (0.27, 0.63)	0.56 (0.40, 0.78)	0.01*
Right	0.44 (0.28, 0.67)	0.43 (0.33, 0.62)	0.39 (0.22, 0.55)	0.55 (0.34, 0.81)	0.03*
Left	0.45 (0.26, 0.66)	0.48 (0.29, 0.66)	0.44 (0.28, 0.69)	0.57 (0.37, 0.79)	0.05*
**RNFL symmetry**					
	77 (46.0, 88.0)	74 (61.5, 81.0)	74.5 (46.5, 84.2)	60.0 (36.0, 75.8)	0.01*

Compared clinical parameters across glaucoma severity statuses; IQR- Interquartile range
*-P-value was derived from comparison of median using Kruskal Wallis test

Online Resource 2 Optical coherence tomography parameters for both patients ' eyes at the initial exam in the Kingston Public Hospital Ophthalmology Clinic were compared across different glaucoma severity statuses

Online Resource 3. Retinal nerve fiber thickness (RNFL) values by quadrant					
	Median (IQR)	Median (IQR)	Median (IQR)	Median (IQR)	P-value
**Superior**					
Bilateral	100.0 (87.80, 113.0)*	98.0 (87.5, 116.0)	102.0 (78.5, 108.0)	81.0 (66.0, 96.5)	<0.001
Right	101.0 (79.0, 111.0)	97.0 (84.8, 122.0)	98.0 (87.0, 11.0)	77.0 (63.0, 103.0)	<0.001
Left	107.0 (87.0, 118.0)	94.0 (82.0, 116.0)	100.0 (71.8, 118.0)	79.0 (65.0, 105.0)	0.002
**Nasal**					
Bilateral	67.0 (60.0, 77.0)	68.50 (62.0, 74.0)	66.0 (58.5, 75.0)	62.5 (56.5, 69.5)	0.003
Right	67.0 (61.0, 77.0)	68.5 (60.8, 77.0)	69.0 (62.0, 79.0)	62.5 (55.8, 71.2)	0.01
Left	66.0 (58.0, 78.0)	67.0 (62.0, 74.0)	64.5 (57.5, 72.2)	62.0 (55.0, 70.0)	0.03
**Inferior**					
Bilateral	107.0 (88.4, 118.0)	99.0 (85.5, 118.0)	99.0 (86.0, 112.0)	77.5 (63.0, 98.0)	<0.001
Right	108.0 (90.0, 119.0)	102.0 (85.0, 119.0)	105.0 (91.0, 120.0)	78.0 (62.8, 113.0)	<0.001
Left	107.0 (93.0, 120.0)	101.0 (86.0, 121.0)	96.0 (68.2, 118.0)	71.5 (60.2, 108.0)	<0.001
**Temporal**					
Bilateral	53.8 (46.5, 63.4)	56.5 (50.5, 64.0)	51.0 (44.0, 58.5)	50.5 (45.0, 58.0)	0.01
Right	55.0 (45.0, 62.0)	56.0 (48.0, 62.0)	49.5 (42.5, 59.0)	51.0 (43.0, 58.0)	0.05
Left	54.0 (47.0, 63.0)	58.0 (54.0, 68.0)	53.0 (44.0, 60.0)	52.0 (43.0, 61.0)	0.01

Compared clinical parameters across glaucoma severity statuses; IQR- Interquartile range
*-P-value was derived from comparison of median using Kruskal Wallis test

Online Resource 3 provides the retinal nerve fiber thickness values by quadrant for both patients' eyes, compared across different glaucoma severity statuses

Online Resource 4. RNFL Thickness by Glaucoma Severity

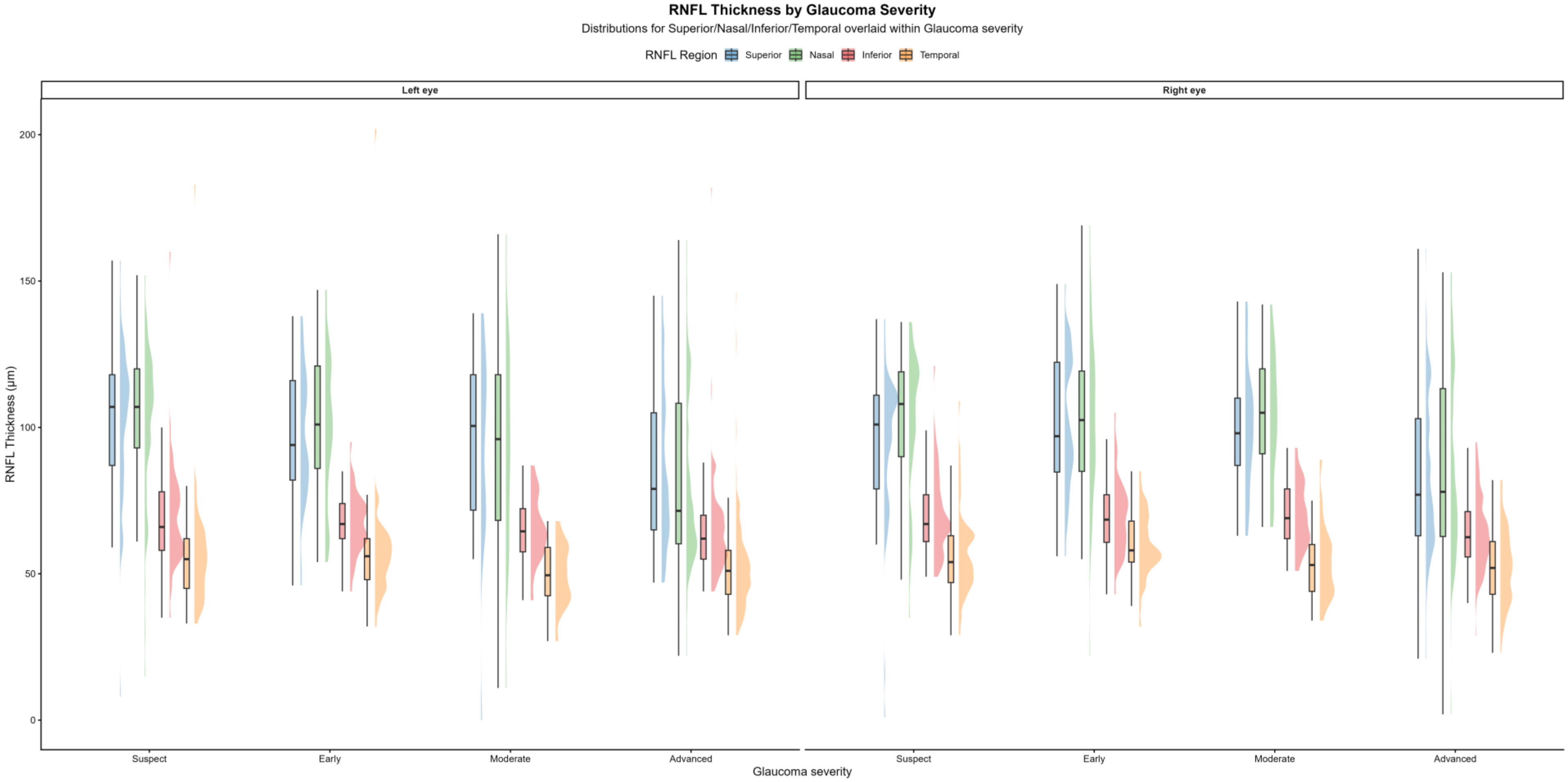

Online Resource 4 shows a raincloud plots distribution of retinal nerve fiber layer (RNFL) thickness across glaucoma severity categories (suspect, early, moderate, advanced) for the left and right eyes. Distributions are displayed for the superior, nasal, inferior, and temporal sectors using raw measurements. The central box represents the median and interquartile range, while the density and individual points illustrate the spread of the data. Left eye, n=236; Right eye, n=234
